# Parvoviruses PARV4 and PARV5 and Hepatitis C Virus

**DOI:** 10.3201/eid1301.060856

**Published:** 2007-01

**Authors:** Jacqueline F. Fryer, Sebastian B. Lucas, David Padley, Sally A. Baylis

**Affiliations:** * National Institute for Biological Standards and Control, Potters Bar, United Kingdom; † Saint Thomas’ Hospital, London, United Kingdom

**Keywords:** Parvovirus, hepatitis C virus, PARV4, letter

**To the Editor:** Parvoviruses are small, nonenveloped DNA viruses that infect both vertebrate and invertebrate hosts. Until recently, parvovirus B19 and adeno-associated viruses, which belong to the genera *Erythrovirus* and *Dependovirus*, respectively, were the only known members of the family *Parvoviridae* that infected humans (*1*). However, 2 recent publications have identified 2 distinct, novel parvoviruses in humans by using the DNase sequence–independent single-primer amplification technique and a related method (*2*,*3*). The first of these viruses, termed PARV4, was observed in a patient with symptoms of acute viral infection syndrome after high-risk behavior for infection with HIV-1, although the patient was subsequently confirmed as negative for HIV-1 (*2*). The second parvovirus was identified in respiratory samples from children with lower respiratory tract infections and termed human bocavirus (*3*).

Parvovirus B19 is a frequent contaminant of plasma pools that are used in the manufacture of blood products, which results in high viral loads in pools and viral transmission in recipients of clotting factors (*4*). We identified PARV4 in such pools (*5*), albeit at a lower frequency and titer than parvovirus B19, when parvovirus B19 was not excluded by screening with nucleic acid amplification techniques. Sequence analysis identified a second genotype of PARV4, which we have termed PARV5, that shares ≈92% nucleotide identity with PARV4 (*5*).

PARV4 was originally identified in a plasma sample from a homeless, injection drug user with fatigue, night sweats, pharyngitis, neck stiffness, vomiting, diarrhea, arthralgia, and confusion (*2*). This person was coinfected with hepatitis B virus. In this study, we looked retrospectively for PARV4 and PARV5 in blood samples from a similar cohort of persons, many of whom were known to be infected with hepatitis C virus (HCV) (as determined by the presence of both HCV RNA and antibodies to HCV), and some of whom were intravenous drug users (IVDUs) (*6*).

Blood samples were collected from 26 cadavers in London and the surrounding area as part of a study to investigate the inhibition of nucleic acid amplification techniques for bloodborne viruses in tissue samples (*6*). The cohort was composed of 10 HCV RNA–positive IVDUs, 8 HCV RNA–positive non-IVDUs, 4 HCV RNA–negative IVDUs, and 4 HCV RNA–negative non-IVDUs (Table). Nucleic acid was extracted as previously described (*4*) by using the MagNA Pure LC instrument (Roche, Basel, Switzerland). PCR was performed with primers specific for the second open reading frame (ORF2) in the PARV4 genome (*2*), which is homologous to the VP1 capsid of parvovirus B19. Primers PVORF2F (5′-AGGAGCAGCAAACAAACTCAGAC-3′) and PVORF2R (5′-TCCTTCATCGCGGCTGTCACTAA-3′) amplify a 268-bp region of ORF2 (nucleotides 2710–2977, GenBank accession no. AY622943). The PCRs were performed and analyzed as previously described (*5*). The assay is highly specific (no cross-reactivity with parvovirus B19) and sensitive (detects 5–10 copies of PARV4 virus DNA per reaction).

**Table:** Analysis of 26 cadavers for parvoviruses PARV4 and PARV5*

Group	No. positive/no. tested	
	PARV4 and PARV5 in HCV RNA–positive cadavers	PARV4 and PARV5 in HCV RNA–negative cadavers
IVDUs	3/10	0/4
Non-IVDUs	0/8	0/4

*HCV, hepatitis C virus; IVDUs, intravenous drug users.

PCR products were cloned, sequenced, and compared with the prototype PARV4. Two blood samples were positive for PARV4, and a third sample was positive for PARV5, with 99%–100% nucleotide identity. These positive samples were from HCV RNA–positive IVDUs (Table). The titer of PARV4 and PARV5 DNA in the positive samples was low and did not exceed >700 copies/mL of plasma, as determined by using a consensus TaqMan assay (J. Fryer, unpub. data). None of the other blood samples tested was positive for PARV4 and PARV5, including those for persons who were HCV RNA negative and not IVDUs.

In our previous study (*5*) of >130 fractionation pools (composed of thousands of units from screened healthy donors) for PARV4, the only positive pools were from North America and no European pools were positive for PARV4 or PARV5. These viruses may be present in such pools but diluted to undetectable levels. In the present study, PARV4 and PARV5 have been identified in blood samples obtained from persons from the United Kingdom. For parvovirus B19, there is evidence of persistent virus infection, at low levels, in bone marrow of previously exposed persons (*7*) and in plasma of immunocompromised and immunocompetent persons (*8*,*9*). There is also evidence for the lifelong persistence of parvovirus B19 (genotypes 1 and 2) in tissues such as skin and synovia (*10*). PARV4 and PARV5 virus genomes share only limited homology with parvovirus B19 (<30% amino acid similarity). Although they have been detected in blood and plasma, nothing is known about the role of these viruses in human disease or their ability to persist in infected persons, healthy or otherwise. Further studies will be required to determine the prevalence of PARV4 and PARV5 in healthy persons compared with its prevalence in those with chronic infections and at high risk, such as IVDUs, and to investigate the nature of persistence of these novel viruses.
